# A critical review of test methods and alternative scientific approaches to compliance and safety evaluation of paper and board for food contact

**DOI:** 10.3389/fchem.2024.1397913

**Published:** 2024-07-11

**Authors:** Angela Störmer, Lisa Hetzel, Roland Franz

**Affiliations:** Fraunhofer Institute Process Engineering and Packaging, Freising, Germany

**Keywords:** food contact materials, paper and board, migration, paper extracts, food safety, dry foods, fatty and wetting foods, migration modeling

## Abstract

Paper and board are widely used as food contact materials. For such sensitive applications, consumer safety regarding the transfer of chemical components and contaminants to the food needs to be established. Such safety assessments are becoming increasingly challenging not only due to intentionally added substances but also non-intentionally added substances. In the European Union, compliance testing and safety evaluation of paper in food contact are largely based on national legislation and standards. The underlying tests are conventional methods, often overestimating and sometimes underestimating the migration into food. In this article, the relevant standard test methods are contrasted with currently available scientific knowledge. The scientific approaches to develop and identify suitable test methods are critically reviewed. Furthermore, theoretical predictions via mathematical modeling, with the aim to realistically simulate transfer to food, are presented and discussed in comparison with available migration studies with foods. Objectives are to (i) summarize the actual scientific knowledge in the field and draw conclusions regarding the potential and limitations of the existing test methods and (ii) identify research gaps toward a better qualitative and quantitative understanding of transport processes of volatile and non-volatile substances from paper and board into foods.

## 1 Introduction

Paper —including cardboard—is increasingly used as food contact material (Technavio, 2023). This is because it seems to almost perfectly serve the objectives of the EU sustainability strategy (Commission European, 2024a; Commission European, 2024b), given that it is based on renewable raw materials and is largely recyclable or compostable. Meanwhile, production of paper is highly energy and water consumptive (CEPI, 2023). The use of recycled fibers reduces the environmental impact (Deshwal et al., 2019; Wellenreuther et al., 2022). However, with recycled fibers, unwanted substances are introduced into the material. These may be such varied that it seems impossible to cover them with relatively simple methods and ensure consumer safety, except for applications with functional barriers like inner bags, barrier coatings, or in direct contact for packing insensitive foods like salt (Biedermann and Grob, 2013; Geueke et al., 2018).

In contrast to plastic food contact materials (FCM), which are subject to a specific and detailed EU legislation (EU, 2023) in a systematic and largely science-based way, paper-based food contact materials still lack such detailed specific regulations at a harmonized European level (Simoneau et al., 2016). One major reason is that for paper, due to the inherent structural and chemical compositional complexity, the knowledge base for proper risk assessment is not as advanced as it is for plastics. Nevertheless, the safety of paper applications in food contact needs to be ensured e.g., in the European Union according to Article 3 of the Framework Regulation 1935/2004 (EU, 2004).

Generally, the transfer of substances from packaging materials to foods must be evaluated to ensure consumer safety and this at best, as realistic scenarios. However, to cover a large number of possible foods as filling goods and avoid analytical difficulties with complex food matrices, simulants and standardized contact conditions are typically used.

Food contact compliance testing and safety evaluation of paper are still largely based on standard methods, which are rather conventional test procedures than simulating transfer to real foods. A systematic and holistic approach similar to plastic food contact materials is still missing but would be needed as a basis for the future EU legislation and framing better rules for the paper packaging industry (Lestido-Cardama et al., 2020; Kourkopoulos et al., 2022). The requirements to ensure consumer safety in food contact applications have been advancing during the last few decades, especially with non-intentionally added substances emerging to the forefront (Koster et al., 2015; Leeman and Krul, 2015; EP, 2016; CoE, 2020; Nerin et al., 2022). Paper food contact materials have been reported in a multitude of scientific articles as potential sources for releasing/migrating chemical contaminants of known and unknown identity and toxicity into the foods when in contact with them. In particular, food contact materials having recycled paper qualities raise concerns (Jickells et al., 2005; Sturaro et al., 2006; Begley et al., 2008; Zhang et al., 2008; Gärtner et al., 2009; Vollmer et al., 2011; EFSA, 2012a; Pivnenko et al., 2015; Canavar et al., 2018; Deshwal et al., 2019; Conchione et al., 2020; Zabaleta et al., 2020; Pan et al., 2021).

Substances with only scarce or no toxicological data can be evaluated as safe only at low migration limits, e.g., by applying Threshold of Toxicological Concern (EFSA, 2012b; CoE, 2020). Test methods—which highly overestimate real migration into foods—may trigger either premature negative evaluations and unjustified non-compliance assessments of paper materials or the need for elaborate and costly migration tests in contact with representative or worst-case foodstuffs themselves (“food prevails”). This risk usually increases with decreasing migration limits.

Overall, there is a high number and variability of applicable tests and evaluation methods in Europe. There is a lot of discussion in the scientific literature, which methods to apply for various purposes, and the related uncertainties and interpretation gaps.

The objective of this article is to (i) provide an overview of available, legally binding, and normative test procedures, including guidance documents in Europe; (ii) compile alternative scientific approaches published in the scientific literature; and (iii) explain and critically discuss the presented test methods and approaches concerning their objectives, potentials, and limitations. The overarching intention is to identify and highlight the resulting conclusions for future research toward a better harmonized rule- and science-based evaluation scheme for paper-based food contact materials.

This paper focuses on simulating the transfer processes to food. Work about methods to detect and identify possibly migrating substances as well as bioassays, although belonging to the area of new alternative approaches, are intentionally excluded from this review.

## 2 A short view on the diversity of paper food contact applications

Paper is commonly used as a packaging material for a wide range of food products, including dry goods (e.g., flour, cereals, snacks, and pasta), baked goods (e.g., bread and pastries), and fresh produce (e.g., fruits and vegetables). Important products for food protection during transport and storage are bags, boxes, and trays. For high-temperature applications, e.g., baking, parchment paper or baking cups for muffins are used to prevent food from sticking to surfaces in commercial and home baking settings and tea bags or coffee filters for hot aqueous contact. In food service areas within the commercial and residential settings, disposable tableware—such as cups, plates, bowls, napkins, and straws—is made from paper. Other applications comprise wrapping materials—including waxed paper—for a variety of food products, such as meat products and sandwiches. Paper can be used as it is or coated with a variety of barrier materials to prevent moisture, oxygen, and aroma compounds from entering or leaving the package and improve grease resistance. Barriers might be coextruded films (e.g., polyethylene), lacquers, or coatings from petrochemical or biobased sources. Finally, labels and tags—applied to food packages or food products for identification or promotional purposes—are often based on paper. A comprehensive overview of paper categories and paper-based food packaging materials can be found in the literature (Simoneau et al., 2016; Deshwal et al., 2019). In conclusion, the use of paper is highly diverse, covering a wide range of products and food contact applications. The paper types and designs depend on the particular application needs and the relevant regulatory standards. Demonstrating or proving the chemical safety of such large variety of applications pose a serious challenge to industrial, contract, and control laboratories, particularly when recycled fibers carrying potentially numerous chemical contaminants enter production lines. Undoubtedly, this situation indicates a need for better science-based methodological support toward the safety assessment of paper food contact materials, as indicated by Grob (2022).

## 3 EU legislation of fiber-based food contact materials and related (supra)national provisions

As with any other food contact material, paper for food contact is subject to the overarching European Framework Regulation (EC) No. 1935/2004 (EU, 2004) and the Good Manufacturing Regulation (EC) No. 2023/2006 (EU, 2006). In short, these regulations lay down general principles to ensure that any food contact material is safe for the consumer and manufactured under quality-controlled conditions. Paper materials are listed in Annex 1 of 1935/2004 as materials, which may be covered by specific EU measures but are not harmonized. Therefore, Article 7 of that Regulation foresees that national provisions can be maintained or adopted by Member States in the absence of EU-specific measures. A total of 10 Member States (Belgium, Czech Republic, Estonia, Greece, Germany, France, Croatia, Italy, the Netherlands, and Slovakia) have set out their provisions and safety criteria in national regulations or recommendations. The latter, such as set out by the German BfR, are legally not binding but have almost the strength of legislation by the force of the market. A summary—including the limits for specific substances—is given in Annex 16 of Simoneau et al. (2016). There is a high variety of rules, requirements, and substance lists with only little congruence between the Member States (Simoneau et al., 2016). The Council of Europe has recently released the general resolution CM/Res (2020)9 on the safety and quality of materials and articles in contact with food (CoE, 2020), which is applicable to non-harmonized materials in EU, giving detailed but not legally binding requirements regarding consumer safety. A specific technical guide on paper and board (CoE, 2021) supplements this general resolution. Annex II gives some restrictions to specific substances occurring within paper. Comprehensive and detailed overviews of the paper EU regulatory situation along with extensive lists of national provisions and measures can be found in the baseline study (Simoneau et al., 2016) and in review articles by Kourkopoulos et al. (2022) and Oldring et al. (2023). A matter of concern in the last decade was the presence of mineral oil components, especially aromatic hydrocarbons (MOAH) in papers with recycled fibers, or from printing inks on food contact materials. The German Federal Ministry of Food, Agriculture and Consumer Protection (BMEL) proposed specific migration limits for MOAH to be undetectable in food and food simulants at detection limits of 0.5 and 0.15 mg/kg food simulant. The respective national decree was not set into force pending a future European measure (BMEL, 2022b;a). Limits for mineral oil components in food are still discussed in EU (EC, 2023).

## 4 Normative framework of standard test methods and guidelines

Supporting and enforcing the European and national legislation for paper, more than 20 standard test methods and procedures are available on European and national levels (Simoneau et al., 2016; CoE, 2021; Oldring et al., 2023). These standards cover a range of test principles (determination of residual content, extraction, and migration), specific target substances or substance groups (such as bisphenol A, anthraquinone, and phthalates), and other paper material parameters, including organoleptic testing, fastness of colors, or optical brighteners.

Testing the transfer of substances from paper includes the step of transfer (migration, gas phase transfer, or extraction) and a specific analytical method for the target substances (BfR, 2015). For simulating the transfer, conventional procedures are usually applied, which shall cover the worst case (BfR, 2015). The four most important and relevant basic test procedures are cold water extract, hot water extract, organic solvent extract, and migration testing using modified polyphenylene oxide (MPPO, poly 2,6-diphenyl-p-phenylene oxide, e.g., Tenax^®^) as a simulant. All procedures are published as European Standards. The extracts are carried out under defined conditions (sample weight, water/solvent volume, and contact time/temperature; Table 1). German BfR recommended slight modifications to increase the intra- and inter-laboratory reproducibility of the water extracts.

**TABLE 1:** Relevant European Standards for the extraction or migration testing of paper

Standard	Short title	Description	Source
EN 645:1993	Cold water extract	Sample, cut or ripped in pieces, extracted with water 10 g/200 mL at 23°C ± 2°C for 24 h, shaking occasionally; the filtrate (filled up to 250 mL) used for analysis	CEN (1993a)
Modification by BfR		Sample cut (not ripped), shaking not necessary, vacuum filtration with a glass fiber filter (1.2 µm) instead of glass drip (10–16 µm), and pressing of the filter cake in case of high water absorption	BfR (2022a)
EN 647:1993	Hot water extract	Extracted with water at 80°C ± 2°C for 2 h. The procedure was similar to that of the cold water extract	CEN (1993b)
Modification by BfR		Similar to that for the cold water extract but no comment on shaking	BfR (2022b)
EN 15519:2007	Organic solvent extract	Sample cut, extraction with ethanol or isooctane 10 g/200 mL contact time and temperature depending on application 2 h or 24 h/20°C (short- or long-term contact) and 2 h/60°C (for hot contact), shaking occasionally; filtrate (filled up to 250 mL) used for analysis	CEN (2007)
EN 14338:2003	Migration using modified polyphenylene oxide (MPPO) as the simulant	Adsorbent MPPO (Tenax^®^) is spread on the surface of the sample (4 g/dm^2^), contact conditions according to application, and solvent extraction of MPPO	CEN (2003)
CEN/TS 14234:2002	Polymeric coatings on paper and board—guide to the selection of conditions and test methods for overall migration	Rapid extraction methods with isooctane and/or 95% ethanol 24 h at 40°C or 50°C depending on the polymer of the food contact layer; for polymer layer thicknesses up to 300 µm	CEN (2002b)

Cold and hot water extracts are considered to simulate direct aqueous food contact; the cold water extract represents aqueous foods and beverages at all applications except hot and baking applications. For these two applications, the hot water extract is used for water-soluble and hydrophilic substances. The organic solvent extract simulates contact with fatty foods by using 95% ethanol and isooctane as solvents. These extractions are carried out with cut samples.

On the contrary, the MPPO test is a migration test, in which the adsorbent MPPO is spread on the food contact surface. MPPO simulates contact with dry foods and at high temperatures with all foods (microwave and baking applications). The test conditions are usually taken from Annex V of the Plastics Regulation (EU) 10/2011 (EU, 2011). The EURL-FCM guideline for testing conditions of kitchenware states examples for a variety of applications (Beldi et al., 2023). For high-temperature applications (microwave and oven), the recommended temperatures vary. EN 14338 gives a maximum temperature of 175 °C for the test but does not give advice on the selection of test conditions. CoE Technical Guide proposes 2 h at 175°C for oven applications and 30 min at 150°C for microwave (CoE, 2021). EURL-FCM guideline—prepared by the EU reference and national surveillance laboratories—recommends conditions up to 2 h at 200°C, depending on the application in the oven and 30 min at 121°C for warming up or defrosting or at 175°C for cooking in the microwave (Beldi et al., 2023). According to German BfR at contact of 2 h at 220°C no degradation for baking papers should occur, and 30 min at 150°C for microwave applications should be applied (BfR, 2015). EURL-FCM guideline distinguishes between coated or treated paper articles, which do not absorb moisture or oil and withstand migration tests based on the conditions from Regulation 10/2011, and other paper articles. For the former, the conditions for plastic materials are given in table 5A of the guideline, and for the latter, the extraction and MPPO tests as described above (table 5B, there). Plastic migration processes have completely different characteristics compared with paper. Consequently, the contact conditions are not necessarily applicable to paper as discussed in depth below in Section 5. In addition to these tests, Council of Europe Technical Guide (CoE, 2021) recommends using 3% acetic acid for estimating the release of metals into acidic foods. Generally, for the contact test conditions, the guide refers to EURL-FCM guideline.

The results of cold and hot water extracts in mg/L are considered conventionally equivalent to migration in mg/kg food (CoE, 2021). CoE Technical Guide states that the real ratio of surface area to the amount of food must be used or the maximum allowable surface-to-food ratio should be declared. By contrast, the EURL-FCM guideline refers to Article 17 of Plastics Regulation 10/2011 and the exemptions for small (<500 g or mL) and very large packages (>10 kg or L) for which the conventional ratio of 6 dm^2^/kg is used. This concept can also be applied to paper (Beldi et al., 2023). Thus, cold and hot water extract results are recalculated to the surface area of the sample and the filling. The newer (or alteratively: more recent) organic solvent standard EN 15519:2007 already requires reporting the results related to the surface.

Contrary to their name, the water and solvent extraction methods are not necessarily exhaustive (BfR, 2015). The extractive power will depend on the type and nature of the target substance (organic-polar, organic-unpolar, or inorganic), its physical–chemical properties, and its interactions with the paper sample. The name “extraction,” which is usually reserved for exhaustive methods, may cause confusion (Oldring et al., 2023). Taking these standards to depict “worst case” migration into food (BfR, 2015), it needs to be stated that these conventional methods might not match the real conditions of use. Confederation of European Paper Industries (CEPI) sees hot water extract testing as a close copy of the intended final use and other extraction tests as mostly overestimating (CEPI, 2019/2021). Although CoE Technical Guide refers solely to these standard tests, German BfR (2015) recognizes this point and recommends a tiered approach using representative real foods in case of doubt or known overestimation or underestimation. Testing results in food have priority for the food regulatory assessment. For plastics, general testing requirements shall cover worst case of food contact applications and be even more severe, as specified in Annexes III and V of EU Regulation 10/2011. Similarly, the methods for paper are intended to simulate transfer into real foods conservatively and are slightly overestimating. However, for plastics, too severe tests can show conformity but not disapprove them. This is the case for alternative methods—the so-called screening methods—but might also be for (overestimating) conventional testing results. EU Regulation 10/2011 in its Article 18 states that “the results of specific migration testing obtained in food shall prevail over the results obtained in food simulant.” This means simulation or prediction of migration should depict the situation with food as closely as possible, ideally matching with the upper bound margin of the determination in the food.

The conventional assumption that the extraction values correlate to migration into food holds a non-negligible conflict potential as migration into food under realistic conditions may differ from the extract results. This becomes obvious for cut samples, which partially disintegrate during extraction. However, the discrepancy may occur in both directions: overestimation and underestimation. The example of perfluoro compounds—which do not fit into the simulant scheme as a worse case (Begley et al., 2008)—is given in BfR guideline (BfR, 2015). Merkel et al. (2018) compared the migration of primary aromatic amines (PAAs) from three paper napkins into four different food categories (wet, dry, acidic, and fatty) with the cold water extract results. In the food category pickled gherkins (aqueous–acidic), cold water seemed to be in seven of nine test cases sufficiently representative or even overestimating with a measured transfer into food (expressed as % of extract) ranging from 62% to 115%, depending mainly on the specific amines, but was severely underestimative in two cases with 224% and 271% (PAA in both cases: aniline). Significantly less, or even no migration could be found into rice (dry), butter cookies (fatty), and cucumber (wet), respectively, with transfer ranging from 2% to 79% and many non-detects. Particularly striking is the difference between the results of cold water extract and butter cookies (fatty foods): only in three of nine test cases, PAAs were detectable in the food and, in which measurable transfer was ranging from 2% to 43% of cold water extract value, i.e., with extreme overestimation by the extract. Cold water extract is the sample preparation method proposed in the recently published standard EN 17163:2019 (CEN, 2019) for testing PAAs.

For the organic solvent extract, the question arises as to whether the surface-related solvent extraction values can be directly understood as representatives of the fatty foods to be simulated. Lestido-Cardama et al. (2020) showed that the solvent extract could be strongly overestimating for lipophilic substance groups like dialkylketones. Solvent extracts in isooctane and dichloromethane were compared with migration into two vegetable oils and three fatty foods (croissant, salami, and two kinds of cheese) under different conditions. The migration into the solvents and oils exceeded the migration into the real foods by roughly four orders of magnitude, suggesting that the solvent and oils are far more extractive than the real foods. It seems that solvent extracts constitute an appropriate and correct tool to determine the migration potential of substances from papers but the obtained results in mass per surface unit cannot be taken as direct measures for considering real transfer to food. However, this practice is still applied. The dialkylketones—for which German BfR has set a migration limit of 5 mg/kg food—are usually directly controlled on the basis of the organic solvent extract.

From the industry side, CEPI released an updated guideline regarding compliance work for food contact paper materials (CEPI, 2019/2021). The guideline is addressed to all participants in the manufacturing chain as well as to the consumers and regulators. CEPI guideline refers to the current standard procedures for testing, including cold water, hot water, and solvent extracts as well as migration into MPPO. It gives recommendations on how to handle limitations of these standards, like the possible overestimation of migration through extracts, e.g., cases in which certain solvent–material combinations could falsely lead to failing results. The solution will be a case-by-case approach, focusing on the risk assessment of the used raw materials in the context of the intended use of the material.

Scientific guidance for theoretically assessing, measuring, and estimating the transfer of mineral oil components was published by German Federation for Food Law and Food Science (Gruber et al., 2019).

## 5 Scientific studies and alternative approaches including migration modeling

### 5.1 Introductory remarks and description of the key challenges

The transfer of a migrant from a packaging material is determined by its mobility/speed (diffusion rate) inside the material and, in the case of semisolid and solid foods, additionally inside the food. The second main parameter is the partitioning between the packaging material and the food, in the case of several layers, additionally between the layers. For compliance testing of plastics, in most cases, liquid simulants are used which shall roughly represent the solubility properties of the food for the migrants. Concerning diffusion and the use of simulants, plastics appear as a simpler matrix compared with paper. The simulant liquids usually do not penetrate the plastic matrix so a kinetic migration test will monitor the time-dependent development of the migrant’s transfer process, ideally in a way that is comparable to the processes occurring in contact with food. Normally migration follows Fickian second law. Therefore, from kinetic data using curve fitting, the physico-chemical key parameters; the diffusion coefficient, D_P_, in polymer; and the partition coefficient polymer-food, K_P/F_, can be derived. Both are fundamental for migration prediction and modeling (Mercea, 2008).

In contrast to plastics, the liquid simulants used in the conventional test methods in the paper sector, i.e., cold or hot water or solvents such as isooctane and 95% ethanol, penetrate uncoated and even often coated paper test samples, thus heavily impairing their functional consistency. This can cause physical disintegration of the paper fiber network. Therefore, the obtained values are rather results of an extraction process and not of a migration mechanism into food. Consequently, this leads in many or even most cases to overestimations because the so-measured values represent an equilibrium between the used (extraction) solvent and the paper material. This differs from real food contact applications, in which the equilibrium is not reached during the contact time. Migration can be slowed down by the diffusion properties of the food itself, especially in the case of semisolid or solid foods. Additionally, the solubility of the migrants in real food may be lower than in simulants, i.e., partitioning is more on the food contact material side. However, tests in food cannot be seen as an alternative to routine testing as they are not possible for all substances and may find their limitations in the analytical feasibility for usually very complex food matrices or even in the choice of representative foods for the intended applications. A way out or at least a supporting tool is the development and use of predictive mathematical migration models, which need to be designed such that they depict reality as closely as possible.

Numerous scientific papers addressed the development of analytical methods for key migrants and contaminants in paper for food contact as well as alternative migration test procedures. The latter aims to define crucial physico-chemical parameters for mass transfer from paper and to find relationships between paper material properties, migrants, test conditions, and migration levels. In the following sections, an overview will be given of published experimental and theoretical scientific approaches toward a better understanding and evaluation of mass transfer from paper food contact materials into foods and their safety in use. This overview is not quantitatively exhaustive but claims to address the most relevant publications with regard to the intention of this article. The conclusions of the studies may depend on the considered migrating substances and their properties, e.g., volatility. The substances used in the respective publications are compiled in the Supplementary Table S1.

### 5.2 Experimental test approaches and key findings

The scientific efforts in the published literature are largely varied and have manifold details. In principle, they can be divided into three major research directions with the following objectives:

(i) Exploring and developing alternative food simulants suitable to mimic food in contact with paper;
(ii) Comparison of migration results obtained from simulated migration testing with realistic migration levels in foods themselves;
(iii) Deriving/defining migration test conditions to better simulate food contact.

#### 5.2.1 Migration into dry foods

Migration into dry foods has been a topic for most of these scientific publications because contact with dry foods represents a major application of paper in food contact. Urbelis and Cooper (2021) published a comprehensive summary review of 162 studies that examined migration into dry foods and its simulants, an important data source. The focus of this study was the extent of migration into dry foods and not specifically from paper packaging but any type of food contact material. However, the largest part is paper relevant. This might correlate with the fact that most packaging for dry foods is paper. This review deals with the analysis of market food products, as well as of food products and food simulants in contact with food contact material, after experimental fortification with known quantities of a migrant. The discussion on testing, information gaps, and remaining questions coincides with this review but is done from a different perspective.

#### 5.2.2 MPPO as a simulant for dry food

MPPO is the official EU food simulant (simulant E) for dry foods in the EU Plastics Regulation (EU, 2023). The development and use of MPPO as a simulant for dry foods go back to the 1990s. Piringer et al. (1993) published the method for the first time as a convenient approach for determining the overall migration at high temperatures such as from non-stick coatings on frying pans and baking papers. The method was also applicable to the determination of specific migration of organic substances from adhesives in paper food contact material (Gruner and Piringer, 1999). Since then, the so-called Tenax method has been increasingly used and has reached a Europe-wide official status with CEN EN 1186:2002-Part 13 (CEN, 2002a) for the overall migration testing of plastic food contact material at high temperature (>100°C) and the implementation of MPPO as food simulant E for dry foods in EU Regulation 10/2011 (EU, 2023). In parallel, the method was standardized for paper food contact material testing (CEN, 2003).

The use and appropriateness of MPPO as a simulant for dry foods were reviewed by Van Den Houwe et al. (2018). The majority of collected data and cited references relate to paper materials. The performance of MPPO as a food simulant for dry foods is discussed based on comparisons with real foods and other food-simulating adsorbents. MPPO simulation compared with the real migration conditions into several foodstuffs (such as sugar, flour, cake and certain pastries, semolina, instant baby formula, milk powder, rice, salt, cereals and even meat, chocolate, sweet matrices, fresh fruits, and vegetables) showed no underestimations of the real conditions. Van Den Houwe concluded that MPPO is suitable as a simulant for dry foodstuffs. Moreover, in their view, the only suitable simulant for the simulation of dry foodstuffs.

#### 5.2.3 MPPO simulant versus dry foods: recycling components other than mineral oil

In 1999–2002, an early and essential scientific initiative was taken by EU Project FAIR CT 98–4318 “Recyclability” within its Section 2 “Paper and Board” (Raffael and Simoneau, 2002; Castle and Franz, 2003). Migration kinetics from 15 different paper sample types were comparatively investigated between dry foods (cookies, flour, milk powder, noodles, salt, semolina, soup powder, sugar, and icing sugar) and MPPO at temperatures ranging from room temperature up to 100°C. A selection of 12 surrogates [such as acetophenone, diphenylether, diisobutylphthalate (DiBP), and diisopropylnaphthalene (DIPN) isomers; Supplementary Table S1] of different chemical structures and volatilities was spiked into the paper samples before testing. Furthermore, partition coefficients between paper and food (simulant) as a crucial mass transfer parameter for migration modeling purposes were determined. Transfer from the paper rapidly reached its equilibrium, depending on substance and temperature, e.g., after 1 h at 100°C and 2–10 days at 23°C. Migration to MPPO was in almost all cases higher than into foods. The final partition ining coefficient between paper and MPPO simulant was always at least one order of magnitude less than the one between paper and foodstuff. Thus, MPPO was found to be more severe than food concerning the adsorption/uptake of migrants from paper; therefore, it was considered to be a good simulant for dry foods. Diisopropylnaphthalene, intrinsically present in the recycled board, displayed a different kinetic behavior compared with the spiked-in experiments, which was explained by “native” DIPN being present in the encapsulated form (from the recycling of carbonless copy papers). This explanation is not necessarily correct because others found differences between “native” and fortified substances also for other components like phthalates (Zülch and Piringer, 2010; Bradley et al., 2015).

Aurela et al. (1999) compared the release of two phthalates (diisobutylphthalate and dibutylphthalate) from paper packages into (crystal) sugar with those into MPPO. The measured migration was similar in sugar stored for 4 months at room temperature and in MPPO stored for 10 days at 40°C or 2 h at 70°C. Alkylbenzenes (C10–C13 alkyl chain) at 30 min at 70°C showed an overestimation factor of 3.8 in MPPO compared with hamburger rolls under the same condition (Aurela et al., 2002).

Baele et al. (2020) observed a strong overestimation for volatile substances (1,3,5-tri-tert-butylbenzene, n-hexadecane, and n-heptadecane) by MPPO (indirect contact, 22°C, 16 weeks) in comparison to starchy, low-fat foods (noodles with and without eggs, wheat, and rice semolina) but similar migration as into chocolate. The difference decreased with decreasing volatility of the migrants. Summerfield and Cooper (2001) compared the migration of dibutylphthalate, diisobutylphthalate, and diisopropylnaphthalene from recycled board into various dry foods and MPPO. In identical conditions (10 days/40°C), migration into MPPO was similar or even lower than migration into rice but similar (diisobutylphthalate) or distinctly higher (diisopropylnaphthalene) when rice were stored for 6 months at 20°C. Diisopropylnaphthalene migrated in similar or even higher amounts in MPPO than in flour and pastry at 40°C. Migration in flour—stored for 6 months at 20°C—was similar to that after 10 days at 40°C.

#### 5.2.4 Mineral oil components (MOSH and MOAH) in market samples

A series of publications (2010–2016) dealt with the migration of mineral oil hydrocarbons (MOH, saturated: MOSH, aromatic hydrocarbons: MOAH) from paper food packaging into foods. The major focus was on dry foods and the underlying mechanisms. The authorship varied but centered around the Cantonal Food Control Lab of Zurich. The starting point was Swiss and Italian market surveys, showing the presence of considerable amounts of MOH in food boxes, which because of the presence of a large fraction of volatile MOH, gave rise to safety concerns owing to their potential to gas phase transfer into the packed foods (Lorenzini et al., 2010). This was confirmed by a follow-up German market survey of 119 samples of dry foods, such as cereals, biscuits, and rice, packed in printed paperboard boxes—with and without internal bags—and intended for longer storage at ambient temperature. In this survey, predominantly saturated hydrocarbons (MOSH), up to approximately C24, were found in dry foods (Vollmer et al., 2011), showing that substances with boiling points up to approximately 400°C can be transferred via gas phase at ambient temperatures from paper into dry foods. This fits with the findings of Jickells et al. regarding transfer from secondary packaging using polar contaminants (Jickells et al., 2005). Even up to C28 is expected to be transferred into dry food like rice in long-term storage (Biedermann and Grob, 2010; 2012). The measurements of replicates from the same samples of the survey were repeated after further 4 months and another 12 months of storage time (Biedermann et al., 2013a). Migration increased from first measurement to third measurement (on average by 60%); however, more than half of the transfer was already found in the first few months. Except for table salt (non-adsorptive food matrix) and noodles (low adsorptive; not specified but most probably without eggs), the migration ranged between 40% and 84% of the potential, after 16 months of storage with semolina, as the most potent adsorbent. The differences between the food types were considered modest. Differences were related more to the packaging materials than to the foods.

#### 5.2.5 Migration studies with mineral oil components under controlled conditions

The above-summarized studies were carried out with market samples from the German survey, in which storage times and temperatures before purchasing were not known (Biedermann et al., 2013a). Additional studies were performed with contact to the food in the laboratory or samples taken directly from the line after filling under controlled conditions.

Dima et al. (2011) explored possibilities for adequate testing of paper party plates, which are covered by a thin polyethylene or polypropylene layer to make them resist liquids from foods. Although there is no direct food contact with paper, including this study here is worthwhile because of the evaluation approach. Furthermore, the thin polymer layers do not act as functional barriers against organic molecules. The authors compared migration of the sum of MOSH and polyolefin oligomers (POSH) of the coating from 16 party plates using an edible oil as a simulant at 70°C, with migration into a variety of fatty foods under foreseeable contact conditions ranging from 60 min to 1 day at room temperature and for some foods with preceding hot contact. The latter was the case for a hot meatloaf, which was freshly fried, placed for 1 h on the plate, and cooled down to room temperature. From the kinetic measurements at 70°C in contact with oil over 120 min, the time point of 30 min was found to reasonably cover the worst case determined in foods. For substances other than mineral oil, edible oil is a complicated analytical matrix in many cases to measure migrants at low levels and due to penetration into paper not suitable for non-coated materials.

In three other studies of this author consortium, the migration of MOSH from paper food contact material into dry foods, such as noodles, rice, and muesli, was investigated (Biedermann and Grob, 2012; Biedermann et al., 2013b; Lorenzini et al., 2013). An important aim was to better understand the transfer mechanisms and the influence of the type and nature of dry foods on migration. Kinetic studies into egg pasta and muesli at five different temperatures (4°C, 20°C, 30°C, 40°C, and 60°C) up to 400 days were performed (Lorenzini et al., 2013). Both food types showed quite similar migration curves with very steep increases at 60°C and with slow migration rates at the low temperatures. However, migration was increasing even after 300 and 400 days. It appeared that all migration points are likely to approach the same or similar migration levels in foods at an infinite time.

In the following study (Zurfluh et al., 2013), “conventional” migration testing of a recycled paper to simulate long-term storage at ambient temperature was studied using MPPO (simulant E). “Conventional” testing was understood to apply test conditions from EU Regulation 10/2011 (for plastics), i.e., 10 days at 60°C; however, 10 days at 40°C were also applied. In addition, polenta (maize semolina) was used at the same conditions as MPPO. The results were compared with the migration into test foods (biscuits containing 18% fat, polenta, noodles, rice, breadcrumbs, and oatmeal) in contact with the same paper packaging material stored for more than 9 months (same experiment as Biedermann et al., 2013b). Simulation with MPPO after 10 days at 60°C led to almost full “extraction” of migratable MOSH (i.e., up to C24), overestimating the maximum migration of MOSH in the real packs by 73%. Ten days of contact with polenta at 60°C gave a similar migration of MOSH as the average of the tested foods. At 40°C, 10 days of contact with polenta underestimated the average migration in the tested foods. Increasing the temperature not only accelerated the migration of given substances but also broadened the range of migrating substances in the direction of lower volatiles. The authors concluded that simulation with MPPO was too overestimative because of the adsorbent and the accelerated conditions of testing. Therefore, MPPO failed in testing the migration of mineral oil from paperboard packaging. The authors questioned the suitability of such simulation for the prediction of long-term migration and proposed determination in paper by defining conventional transfer rates to food (70%–80%).

The food data under controlled conditions in direct paper contact compared with indirect contact (behind polyolefin layers) were separately published (Biedermann et al., 2013b). In addition to MOSH, specific substances like DIPN, phthalates such as DiBP, and several photoinitiators, e.g., benzophenone, were measured as target migrants. This is useful to learn more about the transport mechanisms from paper. For this review, we refer only to the results obtained from direct paper food contact. The foods (choco biscuits, polenta, noodles, rice, breadcrumbs, and oatmeal) were stored for 9 months at ambient temperature with in-between measurements at 2 and 4 months. For the level of migration, there was no severe dependency on the food type (mostly <2 factor), particularly while considering single specific substances rather than the whole group of MOSH. The fastest and highest migration was shown into oatmeal; however, for the aromatic compounds, MOAH and DIPN oatmeal and biscuits were similar. After 9 months, for all six foods, the migration ranged for MOSH <C24 group from 50% to 80% of the initial concentration in paper; however, for specific substances, the ranges were narrower: DIPN 37%–49% (with 26% for breadcrumbs). Migration of DiBP ranged from 0.22 mg/kg food to 0.54 mg/kg food (with an “outlier” of 0.06 for rice and biscuits as the highest) and of benzophenone (as the most prominent photoinitiator) from 24 μg/kg food to 59 μg/kg food (with 6 μg/kg as the lower “outlier” from noodles and oatmeal as the highest). The authors commented this: “Migration seemed to be influenced more by the porosity of the food than by the fat content (for instance, MOSH migration into oatmeal was clearly higher than into fatty biscuits).” This indicates that adsorption rather than dissolution in the fat phase constitutes the driving mechanism for migration of substances with sufficient volatility (according to the authors when compared with C24 or a similar substance). The steepest increase for MOSH was in the first 2 months of storage (approximately 40% of potential, between 22% for noodles and 57% for oatmeal), which increased to 50%–80% after 9 months. As a potentially logical next step, this author consortium studied the difference of more volatile versus non-volatile migrants from paper into dry foods (Eicher et al., 2015). In this study of mechanistic character, data from migration experiments using newspaper as contact with dry foods (rice, polenta, baking mix, and breadcrumbs) and MPPO was reported. The newspaper was chosen because it contained volatile MOSH (<C24) and non-volatile polyalphaolefines (PAO, branched alkanes, characterized by the retention time of the respective n-alkanes) from the printing ink. The authors have differentiated between “direct” (touching) and indirect (gas phase) contacts. They concluded that migration into dry foods via touching contact is not necessarily negligible and could even reach considerable levels. One of the several key experiments was a comparison of 10 and 20 days of contact at room temperature with MPPO, polenta, and rice. The migration focused on three substance classes: MOSH < C24, PAO29, and PAO35, representing increasing molecular weights and decreasing volatility. Migration of MOSH < C24 was 100% on MPPO, 92% for polenta, and 71% for rice. Migration of the non-volatiles PAO29/PAO35 was much lower: 46%/20% for MPPO, 39%/20% for polenta, and 4%/2% for rice. Decreasing the particle size of the polenta from 2 mm to 1 mm led to an increase in PAO migration by factor 2.5 but not of MOSH. Further size reduction to 0.5 mm had only little effect. This was related to the density of contact points in the paper. Migration into MPPO at identical conditions at room temperature (10 days) was in the same order of magnitude as polenta yet lower than baking mix and largely higher than into rice. However, in longer-term (45 days) migration of PAO into rice (higher fat content) passed that of the finer breadcrumbs in contrast to the result after 8 days, in which migration into breadcrumbs was higher. Migration in both rice and breadcrumbs was lower than into polenta. Considering all findings, the authors expressed their concern as to whether MPPO test would be suitable for paper to simulate touching contact: elevating temperature may volatilize non-volatile substances at room temperature, and the particle size of the food may be far from that of MPPO.

The use of surrogates for MOAH while comparing migration testing with MPPO versus the dry foods polenta and couscous was the main topic of research by Jaén et al. (2022). For these kinetic experiments (at 60°C: 3, 6, and 10 days and 70°C: 2 h) cardboard samples were previously fortified with 16 aromatic model substances, such as 2-methylnaphthalene, 2,6-DIPN, and perylene, representing MOAH in a wide range of molecular masses, chemical structures, and most importantly, volatilities (boiling point ranges from 240°C to 467°C). Migration reached equilibrium after 3–6 days at 60°C. For more volatile MOAH substances, the equilibrium levels obtained at 60°C were already reached after 2 h at 70°C. This coincides with the findings of Aurela et al. (1999). In general, the migration values were higher in MPPO than in couscous and polenta, which was highly distinctive for the more volatile surrogates and less distinctive or even the same for the heavier surrogates. The authors concluded that MPPO can be considered as the worst case of the simulation of migration to dry food.

#### 5.2.6 Additional alternative simulants for dry foods

In addition to MPPO (Tenax^®^) and polenta (as a model food), other adsorbents have also been studied as potential dry food simulants: Nerín et al. (2007) performed comprehensive kinetic migration studies on three paper samples with different recycled pulp content using Porapak (a porous copolymer, not further specified in the publication) as a solid-food simulant. Target migrants were DIPN, DiBP, and diethylhexyl phthalate. The test setup was direct (“touching”) contact. Test temperatures were 25°C, 50°C, 75°C, and 100°C with contact times ranging from 5 min to 10 days. In a few selected cases, migration into MPPO and milk powder was carried out for comparison. Porapak was found to allow solid and reproducible measurement of migration kinetics comparable with those obtained with MPPO. Notably, both solid simulants covered reasonably, i.e., with a slight overestimation, and the migration into milk powder occurred at 25°C and 50°C. Fengler and Gruber (2022) studied Sorb-Star as another alternative dry food simulant. Sorb-Star is not porous like MPPO but rod-shaped polydimethylsiloxane (20 mm, ø 2 mm), which is highly adsorptive toward low and medium volatile lipophilic organic compounds. The study compared migration kinetics (at 20°C, 40°C, and 60°C up to 12 days) for MOH using Sorb-Star versus MPPO in “touching” versus “gas phase” contact. The carbon fractions C10–C16, C16–C20, C20–C25, C25–C35, and C35–C50 were investigated to obtain better volatility-resolved information. Furthermore, migration of representative single-substance surrogates for each fraction—alkanes and aromatic compounds—was compared. More polar MOAH migrated slower than MOSH. MPPO in “touching contact” showed the highest values. Under gas phase contact conditions (without direct contact), migration rates into MPPO were lower compared with Sorb-Star. In C25-C35, migration was found only in MPPO-touching contact and Sorb-Star at 60°C for MOSH. The authors concluded that the migration behavior of MOH can be depicted by the use of suitably representative surrogates, which will help ease the analytical tasks. Migration tests with these simulants (MPPO and Sorb-Star) at 20°C and 40°C can cover a wide range of real-life migration processes from paper-based food contact materials into foods, provided that appropriate conditions are chosen.

#### 5.2.7 Impact of humidity

As a potentially important factor, relative humidity (rH)—which could affect the extent of migration and the type of migrants from paper qualitatively—was studied using MPPO by Barnkob and Petersen (2013) and Wolf et al. (2023). In 2013, benzophenone transfer from a paper sample after 30 days at 34°C under three different rH conditions (43%–73%), increased by a factor up to 7.3 with increasing rH. Wolf et al. investigated the effect of rH on the transfer of 59 volatile organic compounds from a paper at different rH setups and temperatures by gas chromatography–mass spectroscopy by comparing peak areas and by sensory tests. Furthermore they compared direct contact of MPPO with the indirect contact with the paper sample. Transfer of volatile substances increased with increasing rH, also depending on the polarity of the substances. The authors concluded and recommended that a defined rH level needs to be established before starting migration or sensory tests to ensure sufficient repeatability and comparability of such tests. In general, touching contact of MPPO with paper led to considerably higher migration values than indirect ones. The influence of humidity from foodstuffs in contact or the environment of storage was also reported by Zülch and Piringer (2010) and Hauder et al. (2013).

#### 5.2.8 Other foods

Bradley et al. (2014; 2015) compared the migration from paper into MPPO with fresh fruit (apples and bananas), potatoes, mushrooms, and raisins, which have different characteristics than typical dry foods such as polenta. Storage tests with fresh foods were performed under realistic time–temperature conditions, e.g., 5 days at room temperature, with raisins and MPPO under standard conditions of 10 days at 40°C. Target migrants were contaminants, intrinsically present in the paper samples such as DIPN or DiBP, as well as surrogates such as benzophenone and dodecane, previously spiked into the paper samples. The major objective of these studies was first to assess the relationship between migration from paper into the foods versus into MPPO and second to study the migration of intrinsically present migrants versus spiked ones. Migration levels depended strongly on the nature of the substance. Migration from spiked P/B samples was more extensive (as a percentage of the available migrants) than that of intrinsic migratable substances, such as DIPN and DiBP. This was explained by a stronger bonding into the fiber network by manufacturing than the spiking process. This difference appears to become relevant for compliance and food safety assessment versus real exposure estimation. In any case, studying spiked samples tends to be conservative. The nature of the substances and of the foods influenced the migration levels much more than the characteristics of the paper samples. Migration into MPPO was up to a factor of 62 (potatoes) but at least by a factor of 10 higher compared with the fresh foods stored for 5 days at room temperature; however, it was comparable or only slightly higher compared with raisins, which due to their long shelf-life, were stored at the same time–temperature conditions as MPPO. The authors discussed the potential use and the limitations of correction factors to correlate MPPO values under standard conditions to realistic food conditions. They concluded that simple correction factors would approximate only the food characteristics but would not reflect the substance-specific nature of chemical migration. Furthermore, they addressed ongoing developments toward a comprehensive migration model for paper that takes into account substance- and food-specific characteristics as modeling parameters (Section 5.3). Correction factors were deduced from migration data and proposed by Castle (2015).

Considering migration into fatty or humid foods, only a few publications are available: dialkylketones into salami, cheese, and croissant (Lestido-Cardama et al., 2020); recycling contaminants into butter (Zülch and Piringer, 2010); and PAAs into humid foods (Merkel et al., 2018).

### 5.3 Predictive migration estimation and modeling

#### 5.3.1 Comparison of modeling in plastics with that in paper

For plastics packaging, migration modeling-based conformity assessment has been officially recognized since 2001 (EU Directive 2001/62/EC), proposed in Article 18 of the current EU Plastics Regulation 10/2011 (EU, 2023) and described in a JRC guideline (Hoekstra et al., 2015). Since then, this tool has been increasingly used by industry, testing laboratories, and authorities (e.g., EFSA and FDA) to evaluate polymer packaging quickly and inexpensively, as well as to cross-check experimental design and results for plausibility. The diffusion of organic substances in plastics generally follows Fick’s second law (Crank, 1975). The plastic layer is considered isotropic and homogeneous with an initially homogeneous distribution of the migrants in the layer. The differential equation from Fick’s law can be solved using numerical simulation (Roduit et al., 2005; Tosa et al., 2008; Nguyen et al., 2013) and is implemented in commercial or free software. The diffusion coefficient(s) D_P_ of a migrant in the plastic layer(s) and partition coefficients K between the layers are required as input parameters. In plastics, D_P_ depends mainly on its molecular size, which allows the estimation of D_P_ using relatively simple formulas (Begley et al., 2005; Piringer, 2008; Welle, 2013; Hoekstra et al., 2015; Mercea et al., 2018). As partition coefficients into food (simulant), if known values are not available, default values for high (K_P,F_ = 1) or low (K_P,F_ = 1,000) solubility in food are employed (Hoekstra et al., 2015).

The assumption of the homogeneity and isotropy of the layer loses its validity in the case of fiber-based packaging materials. Paper mainly consists of cellulose fibers, creating a porous structure, and may contain other additives, fillers, and finishing agents. The transport mechanism can essentially be understood as a sequence of desorption/evaporation steps into the vapor phase of the pores and adsorption/condensation of the migrating substances (Aurela and Ketoja, 2002; Zülch and Piringer, 2010). Nevertheless, various authors considered paper materials as a quasi-homogeneous, isotropic layer and described migration kinetics with the simplified model for plastics.

#### 5.3.2 Models following Fick’s second law of diffusion

The extensive kinetic migration dataset elaborated in EU Project FAIR-CT98-4318 (Raffael and Simoneau, 2002; Castle and Franz, 2003) was used to explore whether the existing migration model for plastics according to Fick’s law could describe the mass transfer from paper while assuming the paper matrix as a homogeneous isotropic layer. The experimental migration curves could be well-fitted based on the effective diffusion constants D_paper_ (understood as the overall diffusion effect within the paper matrix) and partition coefficients K_paper/food_ or K_paper/MPPO_. Effective D_paper_ and K_paper/food_ values were obtained from migration experiments into MPPO and dry foods at temperatures 50°C, 60°C, and 70°C, with eight different paper types spiked and a set of eight migrants. The partition coefficients confirmed that MPPO serves as a more severe adsorbent than foods. From the D_paper_ values, the effective diffusion behavior of the paper samples was found to be similar to LDPE polymer. In general, at temperatures of 40°C and above, migration was dominated by partitioning due to the relatively rapid achievement of equilibrium. At room temperature, diffusion played a bigger role, especially for larger molecules. Therefore, the kinetic model appeared to be more useful in describing short-term contact at ambient temperature and above, e.g., fast foods, and at low temperatures, e.g., chilled and frozen foods. An important finding was that no considerable kinetic differences were noted between the different paper materials, as known for different plastic types.

Zülch and Piringer (2010) developed an adaptation of the plastics migration model for paper. They studied the migration behavior from different paper samples, spiked with model substances and non-spiked with foodstuffs and MPPO as food simulant at −18.5°C and 22°C, and a blotting paper as the acceptor at 40°C. From fitting the migration curve using the plastics multilayer mode of the model (Tosa et al., 2008), they found that in this temperature range transfer from paper will be best described by considering paper as a two-layer system, which is represented by a core layer B1 with relative high diffusion rates and a thin surface layer B2 with different migration behavior. Effective diffusion coefficients were estimated in analogy to the A_P_ value approach for plastics (Begley et al., 2005), based on the molecular mass of the migrating substance up to 400 g mol^−1^. For paper, A_B1_ and A_B2_ are used as specific parameters with constant A_B1_ = 6. A_B2_ value of the virtual surface layer depended on the polarity, humidity in the paper, the water activity of the food, and properties of the migrant ranging between −10 and −1 for contact with dry food and up to 6.0 for contact with butter. This two-layer approach is particularly relevant in the case of low temperatures and migrants with high molecular weight. At high temperatures, the best fit of predicted versus experimental migration data was obtained with a one-layer approach and a common value of A_B1_ = A_B2_ = A_B_ = −2. The authors concluded that the differentiation between the diffusion in B1 and B2 is unnecessary for migrants with low polarity, molecular weights below 350 g mol^−1^ at high temperatures (≥40°C), and high humidity due to the strongly increased desorption rate. With this model, the authors present a full migration model into food for foods like butter, chocolate, pasta, wheat flour, and biscuits at low temperatures (5°C, 22°C). In 2013, the same group (Hauder et al., 2013) published further work to better understand the necessity of the changing the model behavior from a two- to a one-layer approach, depending on the temperature and to refine the model. The specific diffusion behavior in paper and migration modeling from recycled board into dry foodstuffs using n-alkanes with 15–35 carbon atoms and other substances in the board (no spiking) was studied. For the surface region (B2) determining the diffusion rate, the diffusion coefficients of these migrants decreased proportionally to their vapor pressures. Based on these findings, the authors modified the diffusion coefficient equation for B2 with functional consideration of the vapor pressure of the migrants and provided a general migration model for specific and global mass transfer of impurities from the recycled board into the dry food. Barnkob and Petersen (2013) studied the applicability of the paper migration model by fitting the migration of benzophenone from paper at several humidities (40% to >73% rH) at 34°C, using the approach of Zülch and Piringer (2010). The authors found some differences concerning the applicability of the one- and two-layer approaches, which were small within the quality of the fits between both approaches. Han et al. (2016) investigated the migration of photoinitiators into MPPO at 50°C–100°C and derived effective diffusion and partition coefficients by fitting the experimental curves to Fickian second law. Huang et al. (2013) included a term for the paper porosity into their model according to Fickian second law.

#### 5.3.3 Other models

Contrary to the above-described work, Poças et al. (2011) reported that Fick’s second law of diffusion gave poor fits in some cases. They studied the migration of several substances with different chemical functionalities from five different paper materials to investigate the influence of molecular size, chemical characteristics of the migrants, and paper characteristics (such as type, thickness, and recycling content). To fit the migration curves, they explored the potential of Weibull model, which is based on a distribution function triggered by two parameters (scale and shape parameter). It can be empirically applied without the use of physical–chemical parameters, such as D_paper_ and K_paper/food_. Migration from paper was found to be much faster than those from plastics. The volatility and polarity of the migrants determined their transfer into food (simulant) and the losses from the system due to evaporation. The authors concluded that this simple model allows them to describe the pattern of migration curves for a wide range of migrant volatilities. Guazzotti et al. (2015) applied the Weibull model to fit kinetic migration curves obtained from paper, spiked with a series of n-alkanes at 40°C and 60°C in contact with MPPO, confirming that this model can effectively be used to describe a diffusional process of the paper. Another statistical approach to correlate physical–chemical properties with migration behavior was recently published by Jaén et al. (2022) using MOAH surrogates (experimental details are given in Section 5.2). The authors applied multivariate analysis algorithms to correlate and group the migration of model substances and built a partial least squares regression model to predict the worst case MOAH migration. The migration patterns showed strong correlations along with the volatility of the surrogates. The elaborated model was capable of predicting migration values from the physical–chemical substance properties and was a useful tool to be further explored.

Aurela and Ketoja (2002) studied the diffusion of volatile compounds in fiber networks by experimental determination and modeling using random walk simulation, which is based on the porosity of the paper sample and the diffusion speed through the pores—assumed to be the diffusion constant in free air. The compounds were volatile solvents, such as ethanol and butyl acetate. The experimental and modeled effective D_paper_ values matched and were in the range of approximately E-7 m^2^/s (E-3 cm^2^/s) at ambient temperature. The estimate of effective diffusion constants was based only on that of the compound present in free air. Laine et al. (2016) added a term describing sorption to a one-dimensional diffusion equation to simulate migration into MPPO through cardboard in indirect contact and solely through air. The simulated data fitted well with experimental data on migration into MPPO during an indirect contact in an air-filled chamber and after permeation through cardboard using MOSH and MOAH surrogates.

Serebrennikova et al. (2022) described the transport by partial differential equations for transporting within the gas phase of the pores in the paper coupled to those describing the sorption process. The transport processes are determined by complex interactions. Diffusion coefficients and sorption constants cannot be easily derived from experiments but by fitting the parameters of the model to the experimental data (solution of an inverse problem). The example was the diffusion of dimethyl sulfoxide in a stack of paper (23°C, 50% rH), measured at four time points and spatially dissolved in five paper sheets. For solving the complex differential equations (derived from models for water vapor transport), fitting to the experimental data, and obtaining the parameters, they used physics-informed neural networks (PINNs) and successfully compared with finite element methods. In Serebrennikova et al. (2024), the dimethyl sulfoxide experiment (polar component), extended to 12 weeks, and another one with tetradecane (non-polar) were compared with five different mathematical models, which were evaluated by PINN. Three of them were based on Fick’s law of diffusion, with and without a specific term for sorption and desorption. In addition, pseudo-first-order adsorption and second-order reversible sorption models were included. The Fickian behavior models did not fit the data. The best fit was observed for a pseudo-first-order adsorption model (without desorption from the fiber); however, a further search for a suitable function was called for. In general, the authors concluded that PINNs represent a versatile mathematical tool either to validate or to refute the capability of theoretical models to describe experimental data.

### 5.4 Partition coefficients

Although mainly the diffusion processes and estimation of diffusion coefficients D_paper_ are addressed for the migration modeling, access to other crucial parameter—the partition coefficient K_paper/food_—is also limited. The partition coefficients describe the concentration ratio (mass/volume) in the equilibrium. For volatiles, a promising approach determines the partition coefficients of paper and food or MPPO versus air. From the quotient of both, the partition coefficients K_paper/food_ can be derived. Haack (2006) determined the adsorption isotherms of the volatile model substances such as hexanol and others into paper material (at 40°C–120°C), as well as into the foodstuff chocolate, cookies, and pasta—including MPPO as food simulant at 100°C—and calculated partition coefficients from the data. For hexanol, butyl acrylate, nonanal, and diphenyl oxide, K_paper/food_ values for the three foods ranged from 0.03 to 0.88 and K_paper/MPPO_ for MPPO from 0.02 to 0.08, being strongly on the food side. For butanol, K_paper/food_ was between 1 and 4.3 for foods and K_paper/MPPO_ was 3.3. Overall, these data demonstrate the high or at least comparable adsorptive properties of MPPO versus dry foods. These K values largely overlapped with those obtained by curve fitting of migration kinetics within FAIR-CT98-4318 (Raffael and Simoneau, 2002; Castle and Franz, 2003). Triantafyllou et al. (2005) determined partition coefficients of additional substances between paper and air at 70°C and 100°C. Migration kinetics at these temperatures into Tenax (Triantafyllou et al., 2002) and semolina, instant baby cream, and milk powder (Triantafyllou et al., 2007) up to equilibrium are reported from this group; however, partition coefficients were not calculated.

Within the German research project IGF 19016N (Fengler et al., 2019), partition coefficients for mineral oil components (MOSH and MOAH) were determined and published in the guideline document (Gruber et al., 2019). Partition coefficients of MOSH and MOAH between paper and food range between 1,000 (crystalized sugar or honey) and 1 (chocolate or chopped nuts).

## 6 Discussion

From the published results, key findings, and interpretations summarized under Sections 4 and 5, several discussion points arise, which are presented in the following, concise way:

Standard methods to estimate the transfer to foods are extracts with water (cold and hot), solvents (ethanol and isooctane), and migration testing into MPPO. From a physical–chemical point of view, the extracts determine concentrations at partitioning equilibrium in water or solvent. These standard methods have been found to hold potential for overestimation and, in some cases, underestimation of migration into foods, either due to differences in partitioning coefficients of paper versus food or extractant and/or being far away from reaching equilibrium in real applications. Therefore, the extracts cannot be considered to reliably represent the real exposure of the consumer from paper food contact materials in most cases. However, only a little study efforts were made toward wetting or aqueous and fatty contacts.

Migration into MPPO is used for simulating dry foods (EU food contact material simulant E) and heat contact in oven applications. For the latter, there are diverging protocols regarding the test temperature between Member States, EU reference laboratories, and Council of Europe Technical Guide that will need harmonization. However, for the evaluation of these high-temperature applications, no scientific work was found. For a sound decision, not only oven temperatures but also the usually lower temperatures at the direct contact area in real baking applications for large food pieces (cakes and roast) or the shorter times at these high temperatures for cookies should be considered. Furthermore, for setting harmonized test conditions, temperature limitations to MPPO should be taken into consideration. At temperatures of 200°C and higher and in the presence of oxygen from air, MPPO starts to degrade oxidatively, which limits the number of possible reuses after reconditioning.

To experimentally simulate migration from paper into dry foods, there is a broad discussion in the reviewed literature if MPPO is suitable at all, or suitable under which test conditions. MPPO has a highly adsorptive power due to its porosity with a large inner surface and its chemistry. If comparing MPPO and dry foods at identical test conditions, MPPO often highly overestimates migration into foods but may also be in the same range (chocolate) or even less severe (milk powder). However, this conclusion depends not only on the properties of the food but also on that of the substances, mainly the volatility. For room temperature applications, migration may increase over months or years, without reaching equilibrium. Thus, accelerated tests are necessary. However, because of the completely different transport mechanism of gas phase transfer, desorption, and adsorption on the paper fibers in comparison to plastic polymers, an increase in temperature not only accelerates the diffusion rate but also mobilizes substances of lower volatility that would not migrate at detectable amounts at room temperature. The combination of the high adsorptive power of MPPO and acceleration by increased temperature in many cases leads to a high overestimation of migration, as shown in many of the reviewed papers. The conclusions differ: some appreciate the conservative characteristics (Van Den Houwe et al., 2018), whereas others judge MPPO as unsuitable because of the overestimative characteristic (Zurfluh et al., 2013; Eicher et al., 2015).

One approach to overcome this problem is to define a certain time–temperature condition for migration tests (e.g., 30 min at 70°C for short-term contacts), which covers the migration into food in a slightly overestimating way (Dima et al., 2011). Such approaches will need a good statistical basis. Because of the manifold influence factors on migration, a certain time–temperature condition is expected to be valid for a restricted range of substances and food applications. Another approach is to define conventional transfer rates into foods (i.e., typical or worse case percentage of concentration in material) and analyze the paper material (Zurfluh et al., 2013). Such a concept ignores the influence of the material thickness on the transfer rate. Furthermore, and more importantly, it will impede developments for the implementation of barrier properties within the papermaking process. Reduction factors applied to the result of migration into MPPO are a further possibility (Castle, 2015). From comparative data of migration into certain food groups or storage applications, typical overestimating factors of MPPO test were derived and conservative factors were defined, which shall be applied for the evaluation of MPPO test results. However, this must be differentiated based on the volatility of the migrating substance and the type of food. Simple reduction factors will be too crude. Therefore, Bradley et al. (2014) proposed further development of modeling as the better solution. Adapting the geometry of the simulant to that of foods using rods instead of fine particulate adsorbents is an experimental way to reduce the differences to real foods (Fengler and Gruber, 2022) or using real foods (Eicher et al., 2015; Van Den Houwe et al., 2018).

Accepted and validated models—which can simulate the migration out of paperboards into various foods considering the properties of the substances, influences of humidity, paper and food properties, and temperature—will be a solution to overcome all these shortcomings of the experimental tests. However, there is a high demand for research. In the words of Nguyen, the mechanisms and relationships are still poorly understood (Nguyen et al., 2017). At room temperature and below, a non-Fickian behavior was mainly observed. Piringer introduced a virtual surface layer to describe the experimental data by Fickian diffusion (Zülch and Piringer, 2010), allowing the use of the same software established for plastics. The diffusion coefficients are estimated from a semiempirical equation using 95% confidence upperbound parameters. This and other statistical approaches (Weibull and partial least square regression) might be applicable ways for conformity testing but would need further exploration on applicability outside the datasets used for establishment of the parameters. The major research focused on relatively volatile substances (Supplementary Table S1). For non-volatiles and transfer mechanisms other than those via the gas phase, only little work is published.

For understanding the impacts of influencing parameters, defining overestimating parameters is not sufficient. Transport in the gas phase of the pores and desorption and adsorption on the fibers need to be considered. These highly complex interactions cannot be simply derived from experiments. However, initial steps are already taken: Hauder et al. (2013) implemented a term for the vapor pressure; Huang et al. (2013) introduced paper porosity in the modeling equation. For volatiles, the random walk simulation in the pores (Aurela and Ketoja, 2002) will be applicable but for less volatiles, adsorption and desorption on the fibers will play a non-negligible role. Computational approaches by multivariate data analysis and neuronal networks, in combination with physical considerations, are promising. These can help identify the interrelations of various parameters and test the applicability of proposed differential equations and boundary conditions to experimental data. However, the tested models in Serebrennikova et al. (2024) could not yet sufficiently describe the processes and demand for further work.

## 7 Conclusion

Numerous scientific attempts have been made—and are still ongoing—to explore the deficiencies are and the alternative scientific solutions to overcome the shortcomings of existing testing approaches and data gaps. The scientific efforts were focused, in the first place, on the transfer from paper into dry foods under two aspects: (i) how and under which time–temperature conditions migration into dry foods could be simulated and (ii) what would be an appropriate model to simulate and predict migration into food.

Aspect (i): MPPO seems to be the most suitable dry food simulant due to its high adsorptive properties, which makes it a more severe test medium than any dry food but in many cases, a too severe one. For specific applications, representative model foods like polenta or adsorbing rods may serve as options. However, when it comes to the choice of time–temperature contact conditions, there is not enough clarity and targeted precision to match exactly or, at least, very closely the food contact application to be simulated. Several options are discussed but a general approach seems to be difficult. Most of them are related to specific substance groups or volatility ranges and applications. It needs to be explored if the humidity conditions in the experiments need to be defined or not, and if yes, then at which level. From a legal compliance point of view, harsh and very severe time–temperature conditions can be selected, but the results are likely to allow only proving but not disapproving compliance and therefore unnecessarily disqualify paper food contact materials. Estimation of exposure would fail anyway.

Aspect (ii): Out of the different modeling approaches, a physical–chemical model based on the knowledge of the underlying mass transport mechanisms and processes, as well as on the determining parameters, such as diffusion constants and partition coefficients, are, in our opinion, the most promising and sustainable approach. However, there is a lack of qualitative and quantitative understanding of the factors and determinants of transport and partitioning processes in and from paper. For compliance evaluations, it will be advantageous if the plastics model can serve as the format to be adapted to paper along with the main paper-related characteristics, in which the vapor pressure of migrants plays an important role, as was found in almost all studies. For understanding the processes, models must be more complex. Toward wetting or fatty contact, more research is also needed.

Overall, the published scientific data and collective knowledge in this area, along with modern molecular dynamics science, form a promising solid basis for future work to fill the open data gaps and generate the needed knowledge, thus ending up with a migration model of broad applicability and general acceptance.

## Supplementary material

SUPPLEMENTARY TABLE S1 Substances used in paper migration studies.
