# Supplementary material for: A critical review of test methods and alternative scientific approaches to compliance and safety evaluation of paper and board for food contact
Source: Front Chem. 2024 Jul 11;12:1397913. doi: 10.3389/fchem.2024.1397913 (PMC11269091; doi:10.3389/fchem.2024.1397913)
Supplement: Supplementary file 1 [file Table1.pdf]

Supplementary Material

A critical review of test methods and alternative scientific approaches to compliance and safety evaluation of paper and board for food contact

Angela Störmer*, Lisa Hetzel, Roland Franz

Fraunhofer Institute Process Engineering and Packaging, 85354 Freising, Germany

SUPPLEMENTARY TABLE S1: Substances used in paper migration studies

Substance	CAS	MW	References
Aromates and alkyl aromates			
Toluol	108-88-3	92	10A
Styrene	100-42-5	104	5
o-Xylene	95-47-6	106	6, 9, 10B, 20, 22
1,3,5-Tri-tert-butyl-benzene	1460-02-2	246	3, 17
Phenyldodecane	123-01-3	246	3, 8
1,3-Diphenylpropan	1081-75-0	196	8
9,9′ -Dimethylfluorene	4569-45-3	194	1
Naphthalene	91-20-3	128	6, 9, 20, 21,22
1-Methylnaphthalene	90-12-0	142	1
2-Ethylnaphthalene	939-27-5	156	8, 16
2-Methylnaphthalene	91-57-6	142	1
2,6-Dimethylnaphthalene	581-42-0	156	1,
2,6-Diisopropylnaphthalene	24157-81-1	212	1, 3, 4, 10B
2,7-Diisopropylnaphthalene	40458-98-8	212	8
Diisopropylnaphthalenes, isomeric mix	38640-62-9	212	9, 13, 15, 17, 18, 20, 22,23
Biphenyl	92-52-4	154	1
3,3′ ,5,5′ -Tetramethylbiphenyl	25570-02-9	210	1
4,4′-Di-tert-butyl-biphenyl	1625-91-8	266	3
4-Benzoylbiphenyl	2128-93-0	258	13
Acenaphtene	83-32-9	154	1
9-Methylanthracene	779-02-2	192	8, 16
Ethylanthracene	41637-86-9	206	3
t-Butylanthrancene	18801-00-8	234	17

Substance	CAS	MW	References
Phenanthrene	85-01-8	178	3, 4, 8
3,6-Dimethylphenanthrene	1576-67-6	206	1
1-Methylpyrene	2381-21-7	216	1
Triphenylene	217-59-4	228	8, 16
Chrysene	218-01-9	228	1
Perylene	198-55-0	252	1
Benzo(b)fluoranthene	205-99-2	252	1
Isobutylbenzene	538-93-2	134	19
Alkylbenzenes (alkyl chain C10-C13)		218-260	12
Aromates with additonal functional groups			
Benzyl alcohol	100-51-6	108	6
Benzaldehyde	100-52-7	106	3
Acetophenone	98-86-2	120	3, 6, 9, 10B, 20, 21,22
Benzoic acid	65-85-0	122	9, 10B, 20
2,3,4-Trichloroanisole	54135-80-7	211	9, 10B, 20,22,23
Vanilline	121-33-5	152	9, 10B, 20
Triclosan (5-chloro-2-(2,4-dichlorophenoxy)phenol)	3380-34-5	290	4
Bisphenol A (2,2-Bis(4-hydroxyphenyl)propane)	80-05-7	228	4*, 5
Benzophenone	119-61-9	182	3, 4, 9, 10B, 13, 20,21,22,23
4-Methyl benzophenone	134-84-9	196	13
Diphenyl ether, Diphenyloxide	101-84-8	170	9, 10A, 20,22
4-Methyldibenzothiophene	**7372-88-5**	198	1
4,6-Dimethyldibenzothiophene	1207-12-1	212	1
Benzo(b)naphtho(2,1-d)thiophene	239-35-0	234	1
Benzyl 2-naphthyl ether	613-62-7	234	4*
Anthrachinone*	84-65-1	208	10B
Diethylene glycol dibenzoate	120-55-8	314	4*, 13
Dipropylene glycol dibenzoate	27138-31-4	342	4*
2-Phenylmethoxy naphthalene	613-62-7	234	13
1-Hydroxycyclohexyl-phenylketone	947-19-3	204	13
2,2'-Dimethoxy-2-phenylacetophenone	24650-42-8	256	13
Ethyl-4-dimethylamino benzoate	10287-53-3	193	13
2-Ethylhexyl-4-(dimethylamino) benzoate	21245-02-3	277	13
Alcohols			
Ethanol	64-17-5	46	2
1-Butanol	71-36-3	74	2, 10A
1-Octanol	111-87-5	130	3

Substance	CAS	MW	References
1-Dodecanol	112-53-8	186	3
2-Ethyl-1-hexanol	104-76-7	130	6
Acids			
Caproic acid*	142-62-1	116	10A
Abietic acid	514-10-3	303	4*
Aliphatic aldehydes and ketones			
Hexanal	66-25-1	100	10A
Octanal	124-13-0	128	3, 6
Nonanal	124-19-6	142	10A
Dodecanal	112-54-9	184	3
Methyl- ethylketon	78-93-3	72	10A
Di-iso-butyl ketone	108-83-8	142	6
Ethylisopropylketon	565-69-5	100	10A
2-Dodecanon*	6175-49-1	184	10A
Ester			
Acetyl tributyl citrate	77-90-7	402	7, 13
Butylacetat	123-86-4	116	2
Propylacetat	109-60-4	102	10A
Butylacrylat	141-32-2	128	10A
Methyl caproate	106-70-7	130	6
Methylstearate	112-61-8	298	9, 10B, 20,21,22
Di(2-ethylhexyl) adipate	103-23-1	371	13
Alkanes and cylcoalkanes			
n-Octane (C8)	111-65-9	114	6, 10A
n-Decane (C10)	124-18-5	142	19
n-Alkane C10 – C40		142 - 522	8
n-Dodecane (C12)	112-40-3	170	9, 10B, 20, 22
n-Hexdecane (C16)	544-76-3	226	16
n-Heptadecane (C17)	629-78-7	240	17
n-Octadecane (C18)	593-45-3	255	13
n-Eicosane (C20)	112-95-8	283	16
n-Pentacosane (C25)	629-99-2	353	16
n-Pentatriacontan (C35)	630-07-9	493	16
Polyalphaolefin 29		~ 409	14
Polyalphaolefin 35		~ 493	14
Polyalphaolefin 41		~ 577	14
Bicyclohexyl (Cycy)	92-51-3	166	17
1-Cyclohexylbutane	1678-93-9	140	19
1-Cyclohexylheptane	5617-41-4	182	19
1-Cyclohexyldecane	1795-16-0	224	19

Substance	CAS	MW	References
Phthalates			
Dimethyl phthalate	131-11-3	194	3, 6
Diethyl phthalate	84-66-2	222	3, 5, 6
Di-n-propyl phthalate	131-16-8	250	17
Di-n-butyl phthalate	84-74-2	278	5, 6, 7, 9, 10B, 13, 15, 18, 20,21,22
Diisobutyl phthalate	84-69-5	278	3, 4, 4*,5, 13, 18
Di-n-hexyl phthalate	84-75-3	334	6
Di-n-heptyl phthalate	3648-21-3	363	23
Di-n-octyl phthalate	117-84-0	391	5, 6
Di(2-ethylhexyl) phthalate	117-81-7	390	3, 4, 13, 15
Di(isononyl) phthalate	28553-12-0	419	13
Other			
Tetrahydrofuran	109-99-9	72	2
2-Isopropyl-9H-thioxanthen-9-one (ITX)	75081-21-9	254	13

References:

[1] Jaén, J., C. Domeño, S. Úbeda, M. Aznar and C. Nerín (2022). "Migration of mineral oil aromatic hydrocarbons (MOAH) from cardboard containers to dry food and prediction tool." Food Control 138: 109016.

[2] Aurela, B. and J. A. Ketoja (2002). "Diffusion of volatile compounds in fibre networks: experiments and modelling by random walk simulation." Food Additives and Contaminants 19(Supplement 1): 56-62.

[3] Hauder, J., H. Benz, M. Rüter and O. G. Piringer (2013). "The specific diffusion behaviour in paper and migration modelling from recycled board into dry foodstuffs." Food Additives & Contaminants: Part A 30(3): 599-611. (Testsubstanzen waren nur Alkane +DIPN zur Bestimmung der Wiederfindung. Die restlichen Substanzen wurden in einer realen Papierprobe gefunden und ausgewertet)

[4] Zülch, A. and O. Piringer (2010). "Measurement and modelling of migration from paper and board into foodstuffs and dry food simulants." Food Additives & Contaminants: Part A: Chemistry, Analysis, Control, Exposure & Risk Assessment 27(9): 1306-1324. (*not spiked, intrinsic from recycling paper)

[5] Huang, C.-X., D.-D. Duan, M.-M. Yan and S.-F. Wang (2013). "Migration Prediction Model of Residual Contaminants from Food Packaging Paper and its Experimental Verification." Packaging Technology and Science 26: 59-69.

[6] Poças, M. d. F., J. C. Oliveira, J. R. Pereira, R. Brandsch and T. Hogg (2011). "Modelling migration from paper into a food simulant." Food Control 22(2): 303-312.

[7] Wang, Z.-W., S. Gao, C.-Y. Hu and Y.-M. Wu (2015). "Modelling of Migration from Printing Inks on Paper Packaging." Packaging Technology and Science 28(4): 357-366.

[8] Fengler, R., Gruber, L., Kirse, C., and Schmidt, P. (2019). "Abschlussbericht zu AiF 19016 N - Messung und Vorhersage der Migration von Mineralölkomponenten (MOH) aus Verpackungen in Lebensmittel als Beitrag zur Minimierung der Kontamination ". (https://www.fei-bonn.de/gefoerderte-projekte/projektdatenbank/aif-19016-n.projekt).

[9] Castle, L., and Franz, R. (2003). "EU-Project FAIR-CT98-4318 ”Recyclability”. "Programme on the Recyclability of Food Packaging Materials with Respect to Food Safety Considerations - Polyethylene Terephthalate (PET), Paper & Board and Plastics Covered by Functional Barriers". Section 2. Paper & Board. Consolidated project report for the period 01-01-99 to 30-04-02".).

[10] Haack, G. (2006). Untersuchungen zu Wechselwirkungen zwischen Lebensmittelverpackungen auf Kartonbasis mit deren Füllgütern – Bestimmung und Abschätzung von Verteilungskoeffizienten zwischen Kartons und Lebensmittel(simulantien). TU München. https://mediatum.ub.tum.de/603778 (A: volatiles B: semivolatiles)

[11] Aurela, B., Kulmala, H., and Soderhjelm, L. (1999). Phthalates in paper and board packaging and their migration into tenax and sugar. Food Additives and Contaminants 16, 571-577.

[12]Aurela, B., Kulmala, H., and Soderhjelm, L. (1999). Phthalates in paper and board packaging and their migration into tenax and sugar. Food Additives and Contaminants 16, 571-577.

[13] Biedermann, M., J.-E. Ingenhoff, M. Zurfluh, L. Richter, T. Simat, A. Harling, W. Altkofer, R. Helling and K. Grob (2013). "Migration of mineral oil, photoinitiators and plasticizers from recycled paperboard into dry foods: a study under controlled conditions." Food Additives & Contaminants: Part A 30(5): 885–898.

[14] Eicher, A., M. Biedermann, M. Zurfluh and K. Grob (2015). "Migration by ‘direct’ or ‘indirect’ food contact? ‘Dry’ and ‘wetting’ foods? Experimental data for ‘touching’ contact of dry foods with paper and board." Food Additives & Contaminants: Part A 32(1): 110-119.

[15] Nerín, C., E. Contín and E. Asensio (2007). "Kinetic migration studies using Porapak as solid-food simulant to assess the safety of paper and board as food-packaging materials." Analytical and Bioanalytical Chemistry 387(6): 2283-2288.

[16] Fengler, R., and Gruber, L. (2022). Migration of mineral oil hydrocarbons from contaminated paperboard into the food simulants Tenax and Sorb-Star—A comparison. Packaging Technology and Science 35, 603-620.

[17] Baele, M., A. Vermeulen, M. Claes, P. Ragaert and B. De Meulenaer (2020). "Migration of surrogate contaminants from paperboard to foods: Effect of food and surrogate properties." Food Additives & Contaminants: Part A 37(12): 2165-2183.

[18] Summerfield, W., and Cooper, I. (2001). Investigation of migration from paper and board into food-development of methods for rapid testing. Food Additives and Contaminants 18, 77 - 88.

[19] Laine, C., M. Pitkänen, T. Ohra-aho, M. Gestranius and J. A. Ketoja (2016). "Novel Test Approach for Evaluating and Modelling Barrier Properties of Food Contact Materials Against Mineral Oil Contaminants." Packaging Technology and Science 29(11): 571-583.

[20] Triantafyllou, V. I., K. Akrida-Demertzi and P. G. Demertzis (2002). "Migration studies from recycled paper packaging materials: development of an analytical method for rapid testing." Analytica Chimica Acta 467(1-2): 253-260.

[21] Triantafyllou, V. I., K. Akrida-Demertzi and P. G. Demertzis (2005). "Determination of partition behavior of organic surrogates between paperboard packaging materials and air." Journal of Chromatography A 1077(1): 74-79.

[22] Triantafyllou, V. I., K. Akrida-Demertzi and P. G. Demertzis (2007). "A study on the migration of organic pollutants from recycled paperboard packaging materials to solid food matrices." Food Chemistry 101(4): 1759-1768.

[23] Jickells, S.M., Poulin, J., Mountfort, K.A., and Fernandez-Ocana, M. (2005). Migration of contaminants by gas phase transfer from carton board and corrugated board box secondary packaging into foods. Food Additives & Contaminants 22, 768-782
